# Passivation Strategies for Far-Ultraviolet Al Mirrors Using Plasma-Based AlF_3_ Processing

**DOI:** 10.1021/acs.chemmater.5c01881

**Published:** 2025-09-03

**Authors:** Maria Gabriela Sales, David R. Boris, Luis V. Rodriguez de Marcos, James L. Hart, Andrew C. Lang, Benjamin S. Albright, T. Jude Kessler, Edward J. Wollack, Manuel A. Quijada, Scott G. Walton, Virginia D. Wheeler

**Affiliations:** † 41487 NRC Research Associateship Program, Washington, DC, 20001, United States; ‡ U.S. Naval Research Laboratory, Washington, DC 20375, United States; § Catholic University of America and NASA Goddard Space Flight Center (CRESST II agreement), Greenbelt, Maryland 20771, United States; ∥ Nova Research Inc, Alexandria, Virginia 22308, United States; ⊥ 53523 NASA Goddard Space Flight Center, Greenbelt, Maryland 20771, United States

## Abstract

High purity aluminum in its bulk form has intrinsically high reflectance in the far-ultraviolet (FUV) regime and finds utility in astrophysical instrumentation applications. However, bulk Al oxidizes rapidly in the atmosphere, and its native oxide strongly absorbs and severely degrades the observed FUV properties relative to bare Al. Various techniques have been investigated to produce coatings that inhibit aluminum oxide formation and lead to high FUV mirror reflectance. This work examines the development and use of a uniquely modified, hybrid plasma-enhanced atomic layer deposition (PEALD) system to passivate aluminum mirrors with metal fluoride films. This system combines two plasma sources in a commercial atomic layer deposition (ALD) reactor. The first is a conventional inductively coupled plasma (ICP) source operated as a remote plasma, and the second is an electron beam (e-beam) driven plasma near the mirror surface. To establish the operating conditions for the in situ e-beam plasma source, the effects of sample grounding, SF_6_/Ar flow, and sample temperature on resulting AlF_3_ films were investigated. Optimal operating conditions produced mirrors with excellent FUV reflectivity, 92% at 121 nm and 42% at 103 nm wavelengths, which is comparable to state-of-the-art AlF_3_-based passivation coatings and matches that of previously reported ex situ e-beam plasma-processed mirrors. This optimized in situ e-beam process, along with XeF_2_ passivation, is then explored to produce a clean seed layer (unoxidized Al surface) for subsequent PEALD of AlF_3_. Both approaches are demonstrated as valid pretreatments before PEALD of AlF_3_, showing a promising pathway for the deposition of other fluoride-based layers, such as MgF_2_ or LiF, with ALD or PEALD.

## Graphical abstract

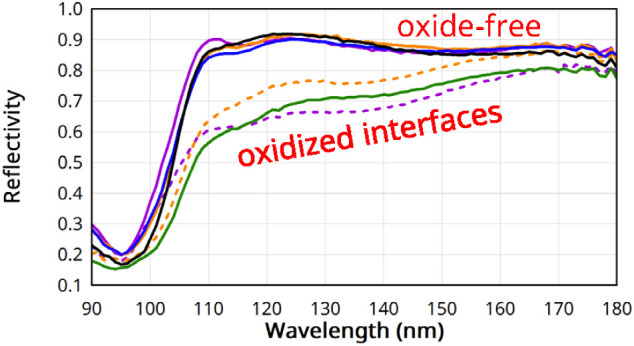

## 1 Introduction

For far-ultraviolet (FUV) astronomy, the high intrinsic reflectance of aluminum has led to its widespread adoption in optical systems and space telescopes. However, the surface of bare Al readily oxidizes and forms a strongly absorbing native oxide layer, which greatly degrades its optical properties at critical wavelengths less than 200 nm.^1^ To prevent native oxide formation, transparent passivation layers are typically used. As a result, current FUV mirrors generally exhibit high reflectivity and low absorptance from the mid-infrared to 120 nm in the FUV.^2,3,4,5,6^ However, technology roadmaps for future space observatories require better sensitivity and higher reflectivity at even shorter FUV wavelengths.^7^

The most promising passivation layers for Al are metal fluoride dielectric films such as MgF_2_, LiF, and AlF_3_, which have intrinsically high transparency above their respective cutoff wavelengths. Each of these fluoride passivation layers comes with performance trade-offs. For instance, the predicted cutoff wavelength for MgF_2_ absorption is 115 nm, while for LiF, it is 103 nm. This lower cutoff wavelength for LiF would extend the useful reflective range in the FUV region; however, LiF is a hygroscopic material and suffers from significant degradation of its optical properties with aging.^8^ The intrinsic cutoff wavelength for AlF_3_ is 105 nm; while this is slightly higher than that of LiF, multilayer films of LiF+AlF_3_ ^9^ or LiF+MgF_2_ ^10^ have been shown to significantly enhance the environmental stability of LiF^9^ while only slightly affecting performance in the FUV.

Atomic layer deposition (ALD) is a thin film deposition technique that provides conformal films with precise thickness control due to its self-limiting and layer-by-layer nature. ALD processes for various metal fluoride thin films have been developed and reported in the literature. MgF_2_ has been deposited by a number of different groups using thermal ALD, at temperatures ranging from 100 to 400 °C.^11,12,13,14,15,16^ LiF ALD has been shown with both thermal ALD at 150–350 °C^17,18,19,20,21,22,23,24^ and plasma-enhanced ALD (PEALD) at 150 °C using an SF_6_ plasma.^25^ Finally, several recipes using thermal ALD^26,27,28,29,30,31,32,33^ and a few using PEALD^33,34^ have been demonstrated for AlF_3_ deposition. Our group also developed a PEALD process for AlF_3_ in which we use trimethylaluminum (TMA) and SF_6_/Ar plasma as our precursor and reactant, respectively.^35^ While AlF_3_ films deposited using this PEALD technique possess excellent material properties (chemistry, purity, and surface roughness), the measured reflectivity of these mirrors is still limited by the poor quality of the AlF_3_/Al interface, which suffers from significant residual oxygen contamination left behind by the native oxide layer.

A method for simultaneously treating the interface and growing an AlF_3_ passivation layer was recently demonstrated using the Large Area Plasma Processing System (LAPPS), which employs an electron beam (e-beam)-generated plasma produced in mixtures of Ar and SF_6_.^36,37^ E-beam-generated plasmas have an easily tunable ion density and very low electron temperature, which provides precise control of the ion flux and energy at the sample surface.^38^ When combined with other plasma sources, the production of ions and reactive neutrals can be decoupled to provide a tunable ion-to-radical flux ratio.^39,40^ E-beam-driven plasmas produced in a large concentration of SF_6_ enable the production of ion–ion plasmas and thus the delivery of fluorine-containing negative ions to adjacent surfaces.^41,42^ This flux of negative ions is critical in removing the native oxide layer and forming a protective AlF_3_ film on Al.^37^ Mirrors processed with SF_6_/Ar e-beam plasma in LAPPS yield 75–95% reflectivity at 108.5–155.0 nm, which are on par or exceed the best physical or chemical vapor-deposited (PVD or CVD) AlF_3_ films reported to date.^36^ The LAPPS e-beam plasma process has the added advantage of being conducted at room temperature. This eliminates the issue of uniformly heating large mirrors during PVD or CVD film growth, which is conventionally done at temperatures as high as 250–300 °C.^3,4,5,9,43,44^ However, since the e-beam plasma process relies on conversion/fluorination of Al into AlF_3_, it can intrinsically only produce AlF_3_ coatings.

In this work, a commercial plasma-enhanced ALD reactor utilizing an inductively coupled plasma (ICP) source was retrofitted to include an e-beam plasma source capable of producing a beam-driven plasma above the sample stage. This is the first known demonstration of using this combination of sources in an ALD reactor. A benefit of ALD/PEALD as a deposition technique is that it is not limited to the production of only AlF_3_. Thus, the main objective here is to use the retrofitted reactor to create an unoxidized, fluorine-containing seed layer in intimate contact with the Al surface prior to subsequent PEALD of metal fluoride thin films. Since the deposition of Al mirrors is performed in a separate chamber, removing the native oxide layer is critical, and several strategies are explored in this work. The first approach was to use an e-beam plasma, both in situ and ex situ, to develop a thin (<10 nm) AlF_3_ layer as a pretreatment before performing conventional PEALD. The second approach involved exposing the Al mirror to XeF_2_ gas in vacuo, immediately following Al deposition. This process served to prevent oxidation of Al by forming a thin (2–4 nm) AlF_3_ layer and was followed by exposure of the passivated Al to the e-beam plasma and PEALD processes. The ultimate goal with these approaches is to provide pathways for multilayer fluoride coatings that can be tailored for enhanced reflectivity while maintaining good stability and reliability.

The findings presented herein are divided into three sections. Section 3.1 discusses the optimization of the operating conditions for the in situ e-beam plasma source in our modified ALD reactor. Section 3.2 compares various techniques to produce AlF_3_ passivation layers, specifically using in situ e-beam plasma, ex situ e-beam plasma, or PEALD. Finally, Section 3.3 discusses strategies to produce an unoxidized Al surface for subsequent AlF_3_ PEALD. All the passivation strategies employed in this work are summarized in Table 1.

**Table 1:** Reference Table of Passivation Strategies

	Processes included				
Strategy number	In-vacuo XeF_2_ passivation	In situ e-beam treatment	Ex situ e-beam treatment	PEALD	Section for results
1		x			[Sec sec3.1], 3.2
2			x		[Sec sec3.2]
3				x	[Sec sec3.2]
4		x		x	[Sec sec3.3]
5			x	x	[Sec sec3.3]
6	x	x		x	[Sec sec3.3]
7	x		x	x	[Sec sec3.3]

## 2 Experimental Section

Initial investigations and optimization of the e-beam plasma conditions were performed on 100 nm Al/10 nm Cr deposited on Si wafers via e-beam deposition. For samples for which reflectivity measurements were obtained, Al mirror substrates were used. These Al mirrors were deposited on soda-lime glass slides using the same coating chamber and process described in a previous report.^10^

For Al mirror processing and AlF_3_ deposition, we used a Veeco Fiji G2 ALD reactor that has been uniquely modified with extra on-axis ports. This allows for the integration of a “hybrid” plasma system, consisting of an e-beam plasma source and an inductively coupled plasma (ICP) source in the same chamber. A schematic of the system is shown in Figure 1, where the radiofrequency (RF)-powered ICP source is located at the top of the chamber and the e-beam plasma is on-axis with the sample surface. The e-beam source is composed of a cold cathode operated to produce a cylindrical electron beam of approximately 2 keV. A permanent neodymium magnet was mounted on the backside of the source flange and cathode, creating a magnetic field to prevent spreading of the e-beam as it propagates across the reactor. The result is a cylindrical plasma located directly above the sample surface. The high-voltage power supply for the e-beam plasma was operated at voltages between 1.5 and 3.1 kV and currents between 6 and 14 mA. For passivation of Al surfaces, the e-beam plasma was ignited in a mixture of SF_6_ and Ar gas, and the sample reference voltage, gas flows, and temperature were all varied and studied in this work. Characterization of the charged particles in the plasma was performed using an Impedans Langmuir probe system with a platinum probe tip. Atomic fluorine density within the ICP was characterized with optical emission spectroscopy (OES) using an Ocean Insight HR2000+ modular spectrometer equipped with a 1200 g/mm grating, which allows 0.1 nm spectral resolution. For samples processed with ex situ e-beam plasma, the optimal operating conditions described in a previous report were used.^36^

**Figure 1:**
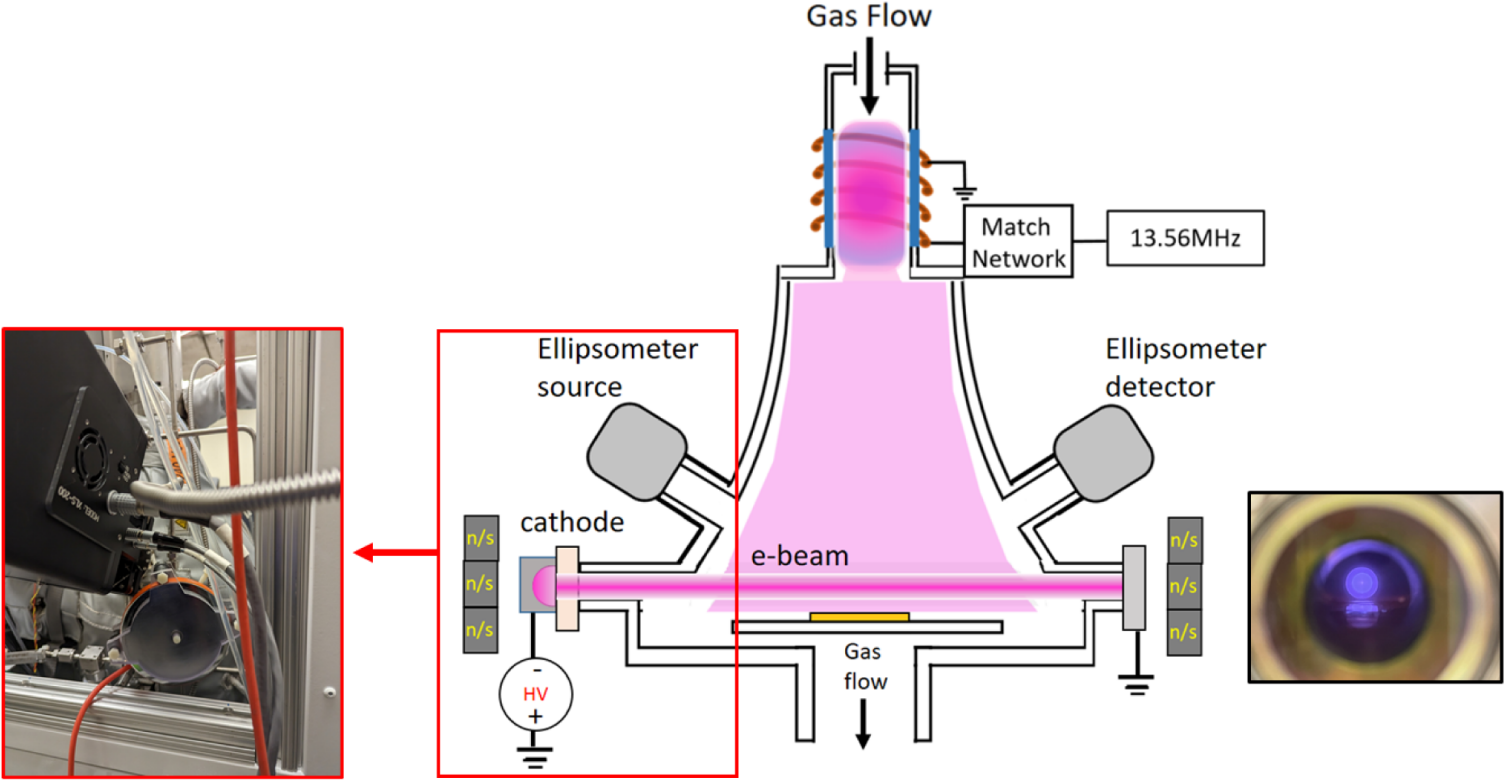
Schematic of the hybrid plasma atomic layer deposition reactor. Pictures shown are of the permanent magnet mounted on the system on the same port as the cathode, and the e-beam plasma glows as seen from the opposite window.

The PEALD recipe for AlF_3_ used in this current study was based on prior optimization work.^35^ Trimethylaluminum (TMA) was used as the Al precursor, and the reactant ICP plasma was produced in a 25/125 sccm mixture of SF_6_/Ar operated at 100 W. The TMA pulse time was 0.08 s, the SF_6_/Ar plasma pulse time was 5 s, and all purges were kept at 8 s. The deposition temperature during PEALD was 150 °C. In combining an e-beam plasma pretreatment (in situ) with PEALD of AlF_3_, as discussed in Section 3.3, there is an approximate 10 min gap between turning off the e-beam plasma source and the first PEALD recipe pulse, during which the sample is held at temperature (150 °C) and under high-vacuum conditions in the ALD reactor. When e-beam plasma pretreatment is performed ex situ in the Large Area Plasma Processing System (LAPPS), the gap between the end of the e-beam plasma exposure and the start of the PEALD recipe is approximately 30 min, majority of which is spent with the sample exposed to ambient temperature and pressure during its transport to the ALD reactor.

To measure the thickness of the resulting AlF_3_ films, spectroscopic ellipsometry (SE) was performed either ex situ using a visible Woollam alpha-SE or in situ using a deep ultraviolet (DUV) Woollam *M*-2000 spectroscopic ellipsometer. Film morphology was measured using a Bruker FastScan atomic force microscope (AFM) operated in the tapping mode, and root-mean-square (RMS) roughness values were calculated from 5 μm^2^ images using NanoScope Analysis software. A Thermo Fisher Scientific Nexsa X-ray photoelectron spectroscopy (XPS) tool, equipped with a monochromated Al Kα X-ray source, was used to characterize the composition and chemistry of the AlF_3_ samples. XPS depth profiling was performed using a monatomic Ar^+^ sputter gun operating at 3 kV with low current settings, which enables gentle etching through the AlF_3_ films to probe the AlF_3_/Al mirror interface.

Reflectivity and transmission of the coated mirrors in the FUV range were measured with a McPherson Vacuum Ultraviolet (VUV) 225 spectrophotometer (mcphersoninc.com). This spectrometer has a one m long high-vacuum monochromator with a 1200 lines/mm grating operating at near-normal incidence in the spectral range from 30 to 325 nm. The spectrometer is equipped with a windowless hydrogen-purged light source, which provides discrete emission lines and/or a continuum, depending on the gas fed and gas pressure parameters. The detector consists of a photomultiplier cathode tube connected to a light pipe for feeding the light signal coming out of the monochromator. The light pipe has a fluorescent high-quantum-efficiency coating of sodium salicylate that is used to convert FUV radiation into visible light. Absolute reflectance and transmittance were obtained by alternately measuring the incident intensity from the hydrogen lamp and then the intensity of the light beam reflected off and transmitted from the sample. Reflectance measurements were performed at 10° from the normal incidence.

To prepare samples for electron microscopy, the sample surface was first protected by using a Sharpie marker, providing an ∼500 nm overlayer. This is an important step since AlF_3_ can be damaged under normal scanning electron microscope imaging conditions.^45^ Next, lift-outs were prepared by using a Thermo Fisher Scientific Helios G3 focused ion beam (FIB). Thinning in the FIB was performed with 5 kV Ga ions, and subsequent final thinning was performed with 1 kV Ar ions in a Fischione nanomill. Scanning transmission electron microscopy (STEM) measurements were performed in a Nion UltraSTEM 200X operated at 200 kV with a 30 mrad convergence semiangle. Electron energy loss spectroscopy (EELS) measurements were collected on a MerlinEELS electron counting detector. To minimize beam damage, the probe current was set to ∼10 pA. For STEM-EELS spectrum images, a large pixel size (1–1.5 nm) and a short dwell time (2 ms) were used to limit the electron dose to ∼1000 e^–^/Å.^2^ The sample composition was determined through a Gatan Digital Micrograph using the Al *L*-edge, C *K*-edge, O *K*-edge, and F *K*-edge. The EELS data were averaged over ∼100 nm along the interface to improve the signal-to-noise ratio.

## 3 Results/Discussion

### 3.1 Optimization of In Situ E-Beam Plasma Conditions

Different parameters related to the operation of the in situ e-beam plasma source were investigated, including the effects of sample grounding, the SF_6_/Ar flow fraction, and sample temperature. The effects of sample reference voltage during e-beam plasma processing were examined by comparing the properties of films formed on a grounded sample surface and an electrically isolated or floating surface. Grounding was accomplished with the use of metallic clips contacting the sample surface to the reactor stage. As shown in Table 2, grounding the sample resulted in higher growth rates, a better stoichiometry of the AlF_3_ layer, and a lower O content in the film.

**Table 2:** Comparison of Growth Rate and Chemistry of AlF_3_ Films Deposited on Electrically Floating and Grounded Al Mirror Substrates

Sample Setup	Floating	Grounded
Growth Rate	2.1 Å/min	4.0 Å/min
F/Al Ratio	2.4	2.8
O Content	7.9 at %	4.1 at %

To understand these results, it is worth considering prior work. E-beam-generated plasmas, when produced in electronegative gases like SF_6_, lead to the formation of ion–ion plasmas.^46,47^ In such plasmas, negative ions replace electrons as the dominant negative charge carrier, thus significantly impacting the attributes of the plasma. Of particular importance for processing applications is that the plasma potential collapses, which allows both positive and negative ions to leave the plasma.^48^ It was previously shown that the growth of an AlF_3_ passivation layer is optimized when negative ions (SF_6_^–^ and F^–^) are delivered to the surface using a positive extraction bias.^37^ The samples grown using the ex situ e-beam system in this work employed a bias of +20 V. However, the commercial plasma ALD system used in this work is designed to provide a negative substrate bias, which can only deliver positive ions to the sample surface; this inhibits AlF_3_ formation as shown in Figure S1. In contrast, and as demonstrated by the AlF_3_ film results in Table 2, when the sample surface is floating or grounded, negative ions are successfully delivered to the surface and are available for AlF_3_ formation. The growth of thick AlF_3_ films is thought to proceed via the diffusion of negative ions into the material, where they react with Al_2_O_3_ and Al to form AlF_3._ The process is presumed to be similar to anodization,^49^ where diffusion is driven by the electric field that develops across the dielectric layer. When the substrate is grounded, the voltage difference and resulting electric field across the layer are greater than if the substrate is allowed to float. As such, the efficacy of the process is improved, as shown in Table 2, when the surface is grounded. All samples processed in the hybrid plasma ALD reactor were thus electrically grounded during e-beam plasma exposure. Reactor modifications needed to provide a positive bias to the substrate are under development.

Next, gas flows in plasma were varied. The maximum allowable SF_6_ flow in the current chamber setup is 25 sccm, and this was kept constant, while the Ar flow was varied. SF_6_/Ar flow ratios of 25/25, 25/80, and 25/150 sccm were investigated. Increasing the Ar flow simultaneously reduces the partial pressure of SF_6_ and increases the total flow/pressure. The AlF_3_ film results from the three flow fractions are shown in Figure 2. It can be seen that the lowest growth rate and worst film chemistry (i.e., lowest F/Al ratio and highest impurity concentration) result from the most F-deficient flow fraction and highest total gas flow studied, 25/150 sccm SF_6_/Ar. The best flow fraction was found to be 25/80 sccm SF_6_/Ar, which produced the highest growth rate, a nearly ideal F/Al ratio of 3.1, and the lowest O content (1.9 at %). The negative ion densities obtained from Langmuir probe measurements were 1.5 × 10^15^ ions m^–3^, 5 × 10^14^ ions m^–3^, and 7 × 10^13^ ions m^–3^ for the 25/25, 25/80, and 25/150 cases, respectively. While all flow conditions met the requirements for the production of ion–ion plasmas, as all negative ion densities exceeded the electron density by 2 orders of magnitude, the best results were not correlated to the largest negative ion density. The reasons for this are not well understood but could be related to operating pressure or chamber wall conditions, which can influence the delivery of species to the surface. In the hybrid plasma ALD reactor used in this work, the effects of total flow and chamber base pressure cannot be decoupled, i.e., a higher total gas flow translates to higher pressure.

**Figure 2:**
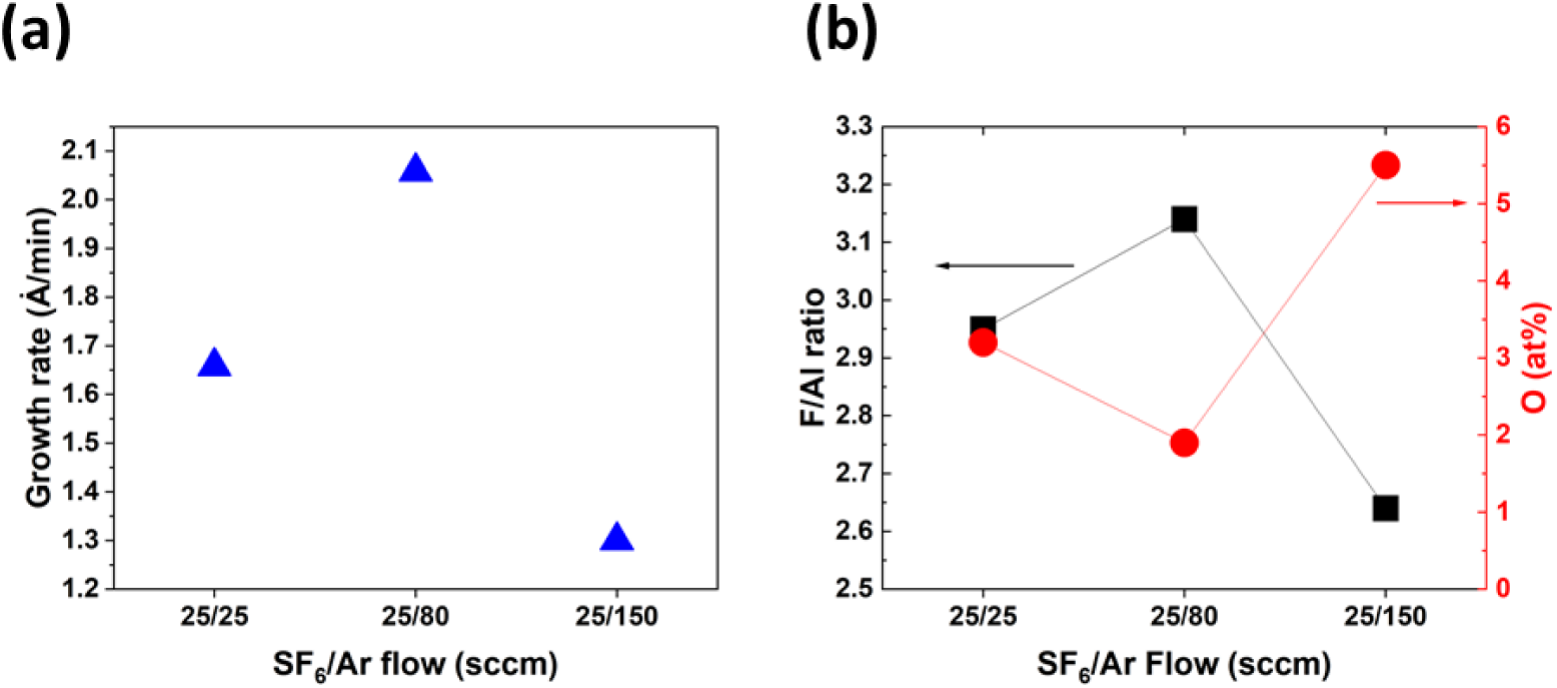
(a) Growth rates obtained from ex situ ellipsometry measurements as a function of SF_6_/Ar flow. (b) F/Al ratio and at % O taken from XPS measurements of the same films.

The last operating condition tested was sample temperature, which was varied between 125 and 300 °C, while keeping all samples grounded and using a constant e-beam plasma exposure time (200 min) and SF_6_/Ar flow (25/80 sccm). Based on the film results, two temperature regions are observed—region I at low temperature (≤150 °C) and region II at high temperature (>150 °C). The two regions are evident in the context of the resulting surface roughness (Figure 3a) and film chemistry (Figure 3b).

**Figure 3:**
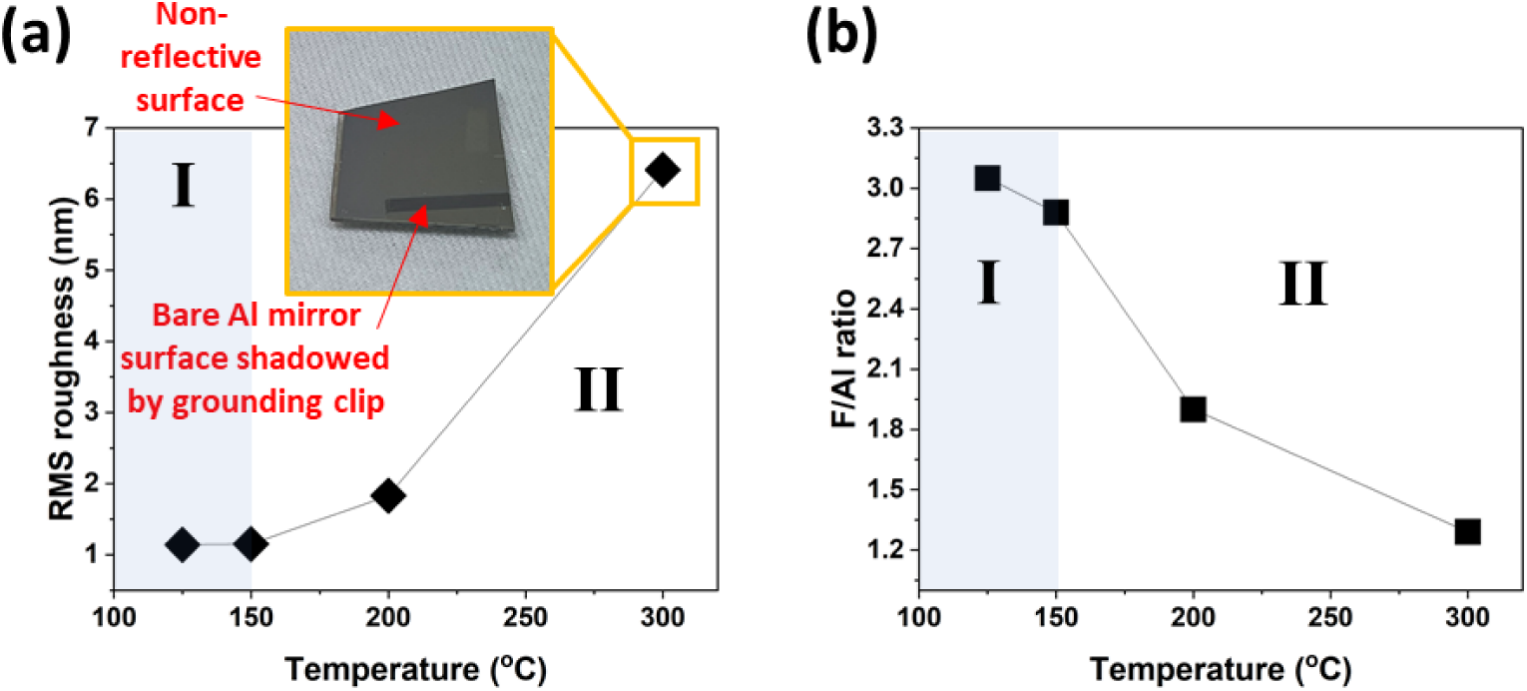
(a) RMS roughness as a function of the processing temperature. The loss of the specular nature of the surface at 300 °C is shown in the inset, where the contrast between the exposed and unexposed (shadowed) regions is apparent. Corresponding AFM images are in Figure S2. (b) F/Al ratio as a function of the processing temperature.

The low-temperature window (region I) produced higher quality AlF_3_ films, with a surface root-mean-square (RMS) roughness close to 1 nm (Figure 3a) and F/Al ratios of 2.9–3.1 (Figure 3b). In the XPS depth profiles, shown in Figure 4a,b, regions with uniform film chemistry are observed at around 150–210 s of etching for the 125 °C sample and at 240–360 s for the 150 °C sample, indicative of the presence of uniform AlF_3_ stoichiometry in those regions of the film away from interfaces. For the 125 °C sample in Figure 4a, the AlF_3_/Al mirror interface is evident where the F concentration decays and the Al concentration starts to dominate, denoted by the dotted line slightly above 240 s of etching. For the 150 °C sample (Figure 4b), this interface was not observed within 360 s of total etching, which shows that the AlF_3_ film produced at 150 °C is thicker than that in the 125 °C sample. Note that for both 125 and 150 °C, the XPS depth profiles reveal a bump in O content within the AlF_3_ film region. These depth profile characteristics are similar to previously reported AlF_3_ films produced in low-F-content e-beam plasmas.^37^ As mentioned above, the SF_6_ gas flow used in this work is maximized in the current system/chamber setup, and further increasing the amount of F in the in situ e-beam plasma is possible but will require system modification.

**Figure 4:**
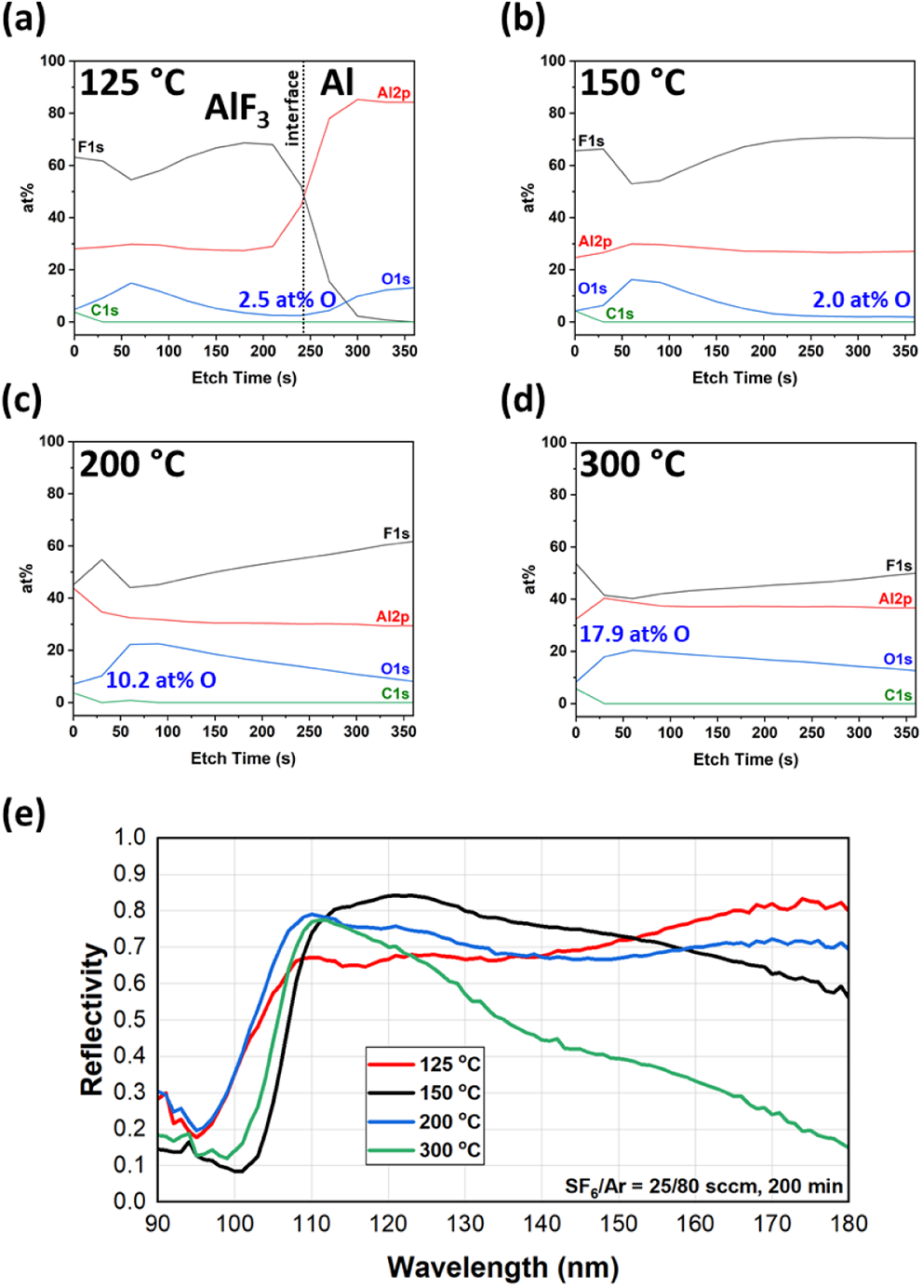
XPS depth profiles showing the relative concentrations of Al, F, O, and C species in samples processed at (a) 125 °C, (b) 150 °C, (c) 200 °C, and (d) 300 °C. The minimum O at % concentration within the AlF_*x*_ film is annotated in the figure for each temperature. (e) Reflectivity as a function of the incident wavelength of samples produced at these processing temperatures.

In region II, surface roughness increases with an increasing deposition temperature. In addition, the specular surface of Al is completely lost at 300 °C, as demonstrated in the inset of Figure 3a. As shown in Figure 3b, very low F/Al ratios (1.3–1.9) are also observed in region II, indicating that stoichiometric AlF_3_ is not produced at these high temperatures. This is further seen in the XPS depth profiles, which are plotted in Figure 4c,d for the 200 and 300 °C samples. At these high processing temperatures, the depth profiles do not reveal any evidence of a uniform AlF_3_ film, as there is no region in which the film composition remains constant within the etch times studied. Several factors may be at play at these increased temperatures. First, higher temperatures would promote diffusion, and it can be observed that the fluorine concentration continually increases with longer XPS etch times, indicative of the presence of fluorine atoms that diffused deep into the Al mirror. Additionally, atomic layer etching (ALE) of Al_2_O_3_, which typically proceeds via fluorination and subsequent removal of the converted AlF_3_ layer, has been reported at temperatures of 155–300 °C.^30,31,32,33,34,35,36,37,38,39,40,41,42,43,44,45,46,47,48,49,50,51,52^ Thus, at the high temperatures in this work (region II), there are likely competing effects between AlF_3_ growth and etching. Lastly, it should be noted that the 10–20 at % O observed in the depth profiles for 200–300 °C (Figure 4c and 4d) is on the same order as the levels of O found in the bulk of the Al mirror substrates (Figure S8), which provides further evidence that fluorination of the oxidized Al surface and conversion/subsequent growth into an AlF_3_ layer did not effectively take place at these temperatures.

The effect of the e-beam processing temperatures on the reflectivity of the mirrors was also investigated. Reflectivity results in Figure 4e show that the optical performance of the 300 °C film is severely degraded at high wavelengths due to significant scattering from its nonspecular surface (Figure 3a, inset). The reflectivity curves of the three other samples all fall within the same range, but as discussed above, more favorable AlF_3_ film characteristics were obtained at lower processing temperatures within region I. Of the two region I films (125 and 150 °C) which have similar qualities of low surface roughness, F/Al ≈ 3, and low O content, the mirror processed at 150 °C was chosen as the most optimal based on measured reflectivity curves. As shown in Figure 4e, the 150 °C sample had the highest reflectivity across the widest range of relevant FUV wavelengths, ranging from 73% to 84% over wavelengths extending from 110 to 150 nm. While this total reflectivity curve is still substandard, potentially due to incomplete removal of the native oxide layer and/or nonideal AlF_3_ film thickness, the 150 °C sample exhibited the largest region of uniform AlF_3_ film chemistry in the XPS depth profiles. As such, based on the cumulative results of this section, 150 °C was used for all succeeding in situ e-beam processed mirrors discussed below.

### 3.2 Comparison of Mirror Passivation Techniques with AlF_3_: In Situ E-Beam Plasma, Ex Situ E-Beam Plasma, and PEALD

This section compares different mirror surface passivation strategies (Strategies 1–3 in Table 1: in situ e-beam, ex situ e-beam, and PEALD only), with particular emphasis on the AlF_3_/Al interface produced. Theoretical calculations suggest that an AlF_3_ thickness of 24–28 nm, depending on the density of the AlF_3_ film, provides maximum reflectivity at the 121.6 nm wavelength (H Lyman α line).^36^ Thus, this was the targeted thickness for all AlF_3_ films compared to that here. Figure 5 shows the measured reflectivity curves for an optimal in situ e-beam-processed mirror (processing parameters discussed in Section 3.1), a standard e-beam plasma-processed mirror in the Large Area Plasma Processing System or LAPPS^36^ (labeled “ex situ e-beam plasma”), and a PEALD AlF_3_ film. The performance of the optimized in situ e-beam mirror successfully matched the state-of-the-art from LAPPS, demonstrating that the e-beam plasma process for mirror passivation^36^ can be adapted into other chambers, such as an ALD reactor. However, the optimal PEALD-only AlF_3_ film, fabricated in the same ALD reactor, has worse performance over the measured wavelength range than either e-beam-only processed mirror.

**Figure 5:**
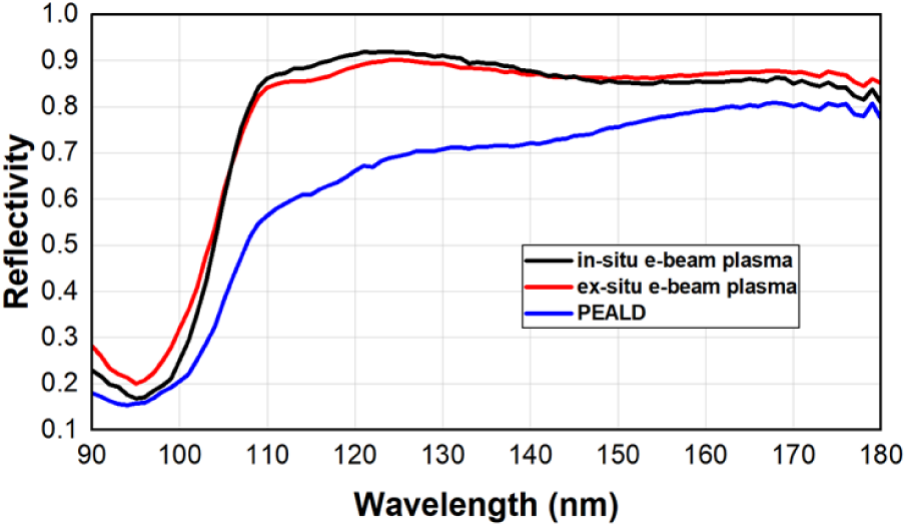
Reflectivity as a function of the incident wavelength of AlF_3_-passivated mirrors processed using different methods (Strategies 1, 2, and 3 in Table 1).

To elucidate the reason for their differences in reflectivity performance, these passivated mirrors were analyzed with AFM for surface roughness, XPS for film and interface chemistry, and STEM for the interfacial structure and film uniformity. The samples used for XPS, TEM, and reflectivity measurements were all processed in each chamber simultaneously to ensure that they all underwent the exact same processing conditions. AFM measurements (images shown in Figure S3) revealed an RMS roughness of 1.81 nm for the in situ e-beam sample, 1.06 nm for the ex situ e-beam sample, and 0.88 nm for the PEALD sample. The roughness of the in situ e-beam film is slightly higher than the best RMS roughness values reported in the previous section; however, this is possibly due to variability in the roughness of the starting mirror surfaces. As shown in Figure 5, high reflectivity is still achieved with the in situ e-beam plasma process despite a slightly increased surface roughness. This suggests that within a certain range of RMS values (i.e., <1 to 2 nm), surface roughness does not have a significant impact on the FUV reflectivity of the mirror and cannot explain the differences between e-beam and PEALD AlF_3_ performance.

In terms of film chemistry from XPS analysis, a relatively flat depth profile, indicative of constant composition and uniform AlF_3_ chemistry, was observed for all three samples. As shown in Figure 6a,d, and g, film regions with uniform chemistry are observed between 0 and 150 s etch time for both e-beam plasma samples and between 0 and 100 s etch time for the PEALD sample. The F/Al ratio is calculated from this film region, and all samples had close to the expected F/Al stoichiometry of 3; specifically, 3.16 for in situ e-beam, 3.17 for ex situ e-beam, and 2.94 for PEALD. Furthermore, the O concentrations within the AlF_3_ film for these three samples were all minimized at 1–2 at %.

**Figure 6:**
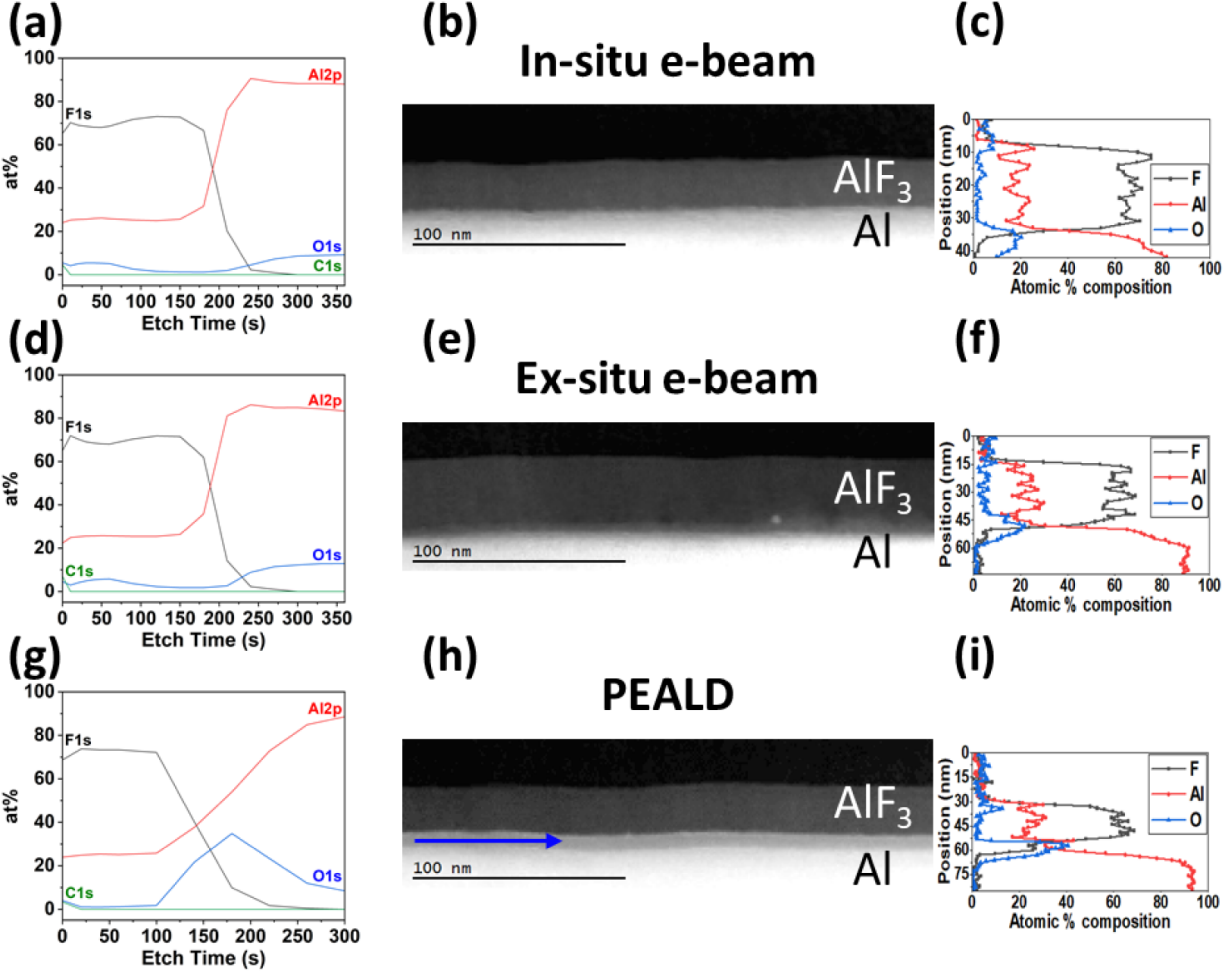
XPS depth profiles showing the relative concentrations of Al, F, O, and C species for AlF_3_ on Al processed using (a) our optimal in situ e-beam plasma operating conditions, (d) standard LAPPS ex situ e-beam plasma conditions, and (g) our optimal PEALD AlF_3_ recipe. Cross-sectional STEM-ADF micrographs and accompanying EELS elemental profiles, respectively, of the (b and c) in situ e-beam, (e and f) ex situ e-beam, and (h and i) PEALD samples. The blue arrow in (h) indicates the interfacial oxide.

The most apparent difference between all the depth profiles is in the PEALD-only sample (Figure 6g), where there is a large bump in the O concentration after etching through the AlF_3_ film, peaking at around 180 s of etching, which corresponds to the interface region between the AlF_3_ film and the Al mirror substrate. This is in contrast to the minimal O content at the interface for both in situ and ex situ e-beam samples in Figure 6a,d, respectively. Quantifying these results, both e-beam plasma processes (in situ and ex situ) produce 1–2 at. % of O at the interface, while the PEALD process results in 35 at. % interfacial O.

Further analysis of this interface was performed with STEM, which was challenging because of surface oxidation and beam damage effects during characterization. STEM-EELS measurements indicate that after FIB lift-out sample preparation, the sample lamella suffers from oxidation, particularly at the AlF_3_/Al interface (Figure S6a), which could explain the interfacial O found in the STEM-EELS cross sections, despite its absence in the corresponding XPS depth profiles for the in situ and ex situ e-beam processed samples. In addition, AlF_3_ is known to take damage during electron beam exposure,^45^ and all AlF_3_ samples analyzed here also showed rapid degradation during imaging, i.e., metallization into Al islands (see Figure S7). To minimize sample damage, the local electron dose was kept at ∼1000 e^–^/Å,^2^ which adversely affected image quality and limited the spatial resolution to 2–3 nm. While any oxidation and electron beam damage during sample preparation and imaging are hard to eliminate, samples of similar thicknesses, imaged under identical conditions, can be compared.

Cross-sectional STEM annular dark-field (ADF) results from AlF_3_ films fabricated using different passivation techniques are presented in Figure 6b,e, and h. The micrographs reveal that all three processes successfully produce a continuous AlF_3_ layer with a uniform thickness throughout. Further, from EELS measurements (Figure 6c,f, and i), the O concentration within this AlF_3_ layer was ≤2 at % for all samples (see also Figure S6b). Note that for the EELS data, carbon is included in the calculation of atomic % composition but is not plotted in the figures for clarity. The AlF_3_ film structure and chemistry are comparable for in situ e-beam plasma, ex situ e-beam plasma, and PEALD samples, and the main differences lie at the starting mirror interface.

At the AlF_3_/Al mirror interface, STEM-ADF imaging shows a clear interfacial layer for the PEALD sample that is approximately 7 nm thick (blue arrow in Figure 6h). In contrast, there is no distinct interfacial layer for the e-beam-plasma-treated samples (given the measurement spatial resolution of 2–3 nm). EELS elemental analysis, shown next to each STEM micrograph, reveals that the O at % concentration at the interface for the PEALD sample (peaking at 41 at %) is about 2× that of the e-beam plasma samples (maximum of 20–22 at %). While these O concentrations from EELS do not exactly match those measured from XPS, due to oxidation of the STEM samples, the same trends are observed. In both cases, the concentration of O measured at the interface is significantly decreased with the e-beam plasma process, whether in situ or ex situ.

The key characteristic of the e-beam plasma process (Strategies 1 and 2 in Table 1) is the removal of the native oxide at the Al interface during AlF_3_ growth, whereas the PEALD process (Strategy 3 in Table 1) does not directly treat the starting native oxide on the mirror and deposits AlF_3_ on the substrate surface as is. The XPS and STEM characterization presented here provide evidence that the e-beam plasma (in situ and ex situ) successfully mitigates at least some, if not all, of the native oxide on the starting Al mirror surface, but that after film growth, both e-beam plasma and PEALD result in comparable AlF_3_ films on top of the mirror. Since the main difference is the interface quality between the AlF_3_ and Al, it can be inferred that the presence of the interfacial oxide layer in the PEALD-processed mirror limits its optical performance (Figure 5).

These results point to the potential of a hybrid plasma ALD system that allows one to combine the benefits of an e-beam plasma for removing native oxides at low processing temperatures with any PEALD process. To this end, the following section focuses on pretreatment strategies for PEALD.

### 3.3 Pretreatment Strategies to Produce Unoxidized Al Surfaces for Subsequent PEALD

This section explores mitigation strategies to minimize the amount of O at the AlF_3_/Al interface since it is critical to achieving the best reflectivity and optical performance of coated mirrors. While an optimized PEALD AlF_3_ recipe is used here for proof of concept, the approaches discussed could be extended to other metal fluoride ALD processes pertinent to FUV mirror applications.

Strategies 4 and 5 from Table 1 are first discussed. For this section, experiments were carried out such that the time between the e-beam plasma pretreatment and the PEALD process was minimized. The PEALD recipe was started either within 10 min (for in situ) or 30 min (for ex situ) of turning off the e-beam plasma source. Within this time frame, the films produced using the in situ process were held under vacuum, while the ex situ films were exposed to the atmosphere. As measured by ellipsometry, approximately 7–10 nm of AlF_3_ was initially grown via e-beam plasma exposure. This was followed by a PEALD of about 13–14 nm of AlF_3_. The total AlF_3_ thickness after completion of both the e-beam and the PEALD processes was 20–24 nm.

AFM analysis shows no significant difference in surface roughness between the in situ and ex situ e-beam pretreatments. This is not surprising because AFM measures the roughness of the PEALD film on top, which was grown using the same procedure/recipe for both the in situ and ex situ cases. It is of note that both films have an RMS value of <1 nm (Figure S4), which is comparable to that of a bare Al mirror (Figure S5). This shows that the e-beam pretreatment and optimized PEALD recipe do not add additional roughness to the mirror surface.

Similar to that above, STEM, EELS, and XPS were used to investigate the films and their interfaces. EELS elemental profiles measured from cross sections of each sample are plotted in Figure 7a,c, in which the AlF_3_/Al interface region is highlighted in yellow. Note that as discussed previously STEM sample preparation and exposure to the electron beam during imaging induce damage to AlF_3_ and may cause an inflated O concentration measurement. As such, STEM-EELS is used here as a qualitative tool for determining O content. Within the limits and resolution of the measurement, no difference in the interfacial O concentration for both in situ and ex situ pretreatments was observed. As shown in Figure 7a,c, both e-beam-pretreated samples have a peak of 22–24 at. % of the atomic weight (at. %) of O at the interface, which is similar to the e-beam-only samples in Section 3.2 (Figure 6c,f). However, both pretreated PEALD films (in situ or ex situ) exhibit a lower interfacial O concentration than a PEALD film without any pretreatment, which, as shown earlier in Figure 6i, had 40 at. % of O at the interface.

**Figure 7:**
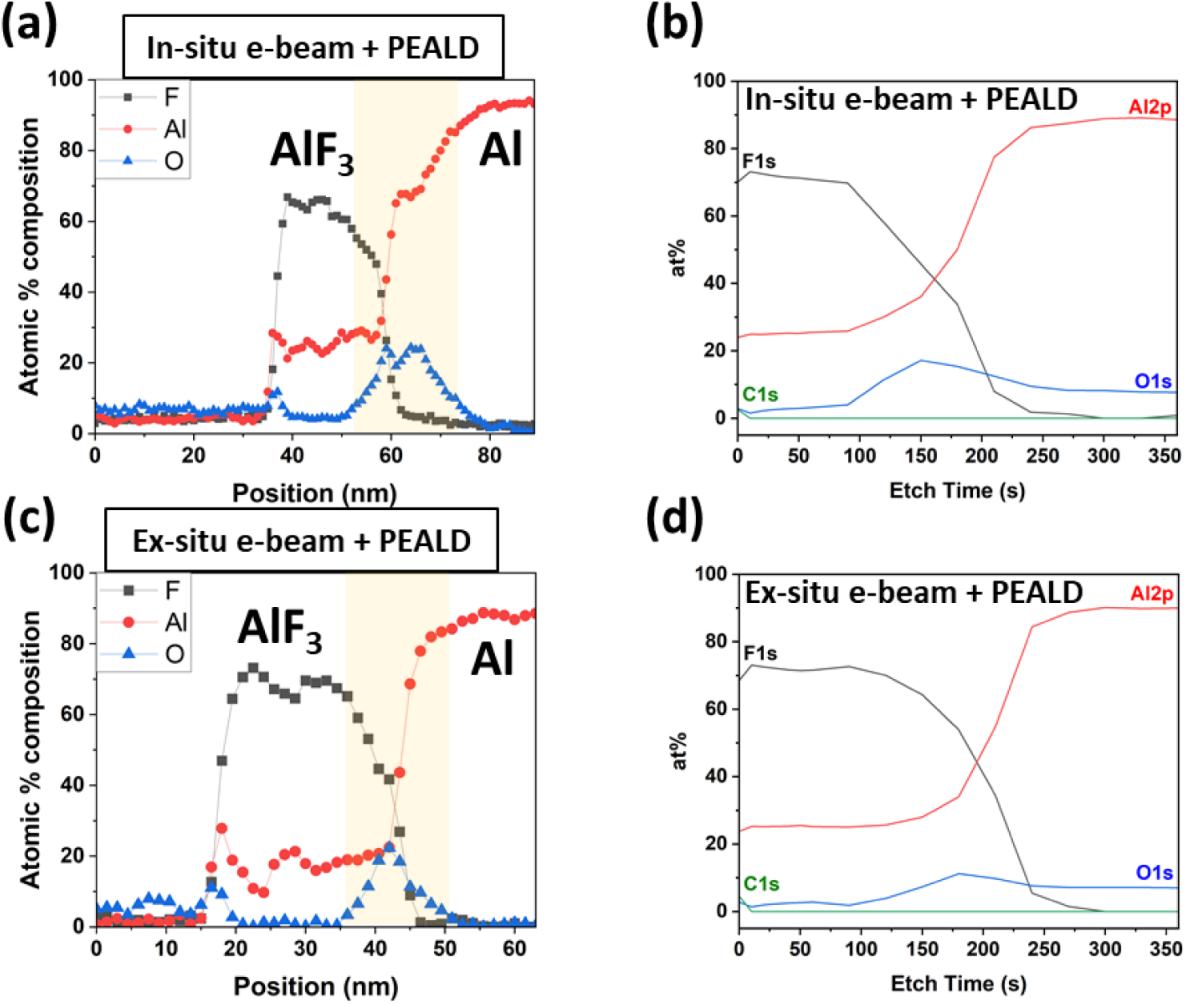
STEM-EELS elemental profiles for F, Al, and O, and XPS depth profiles showing the relative concentrations of Al, F, O, and C species for (a and b) an in situ pretreated PEALD film and (c and d) an ex situ pretreated PEALD film (Strategies 4 and 5 in Table 1). For the STEM-EELS profiles, the 0 nm position starts at the carbon layer deposited on top of the stack to protect the sample during STEM analysis, and the interface region between the AlF_3_ film and the Al mirror is highlighted in yellow. For the EELS data, carbon is also included in the calculation of atomic % composition, but is not shown in the figure for clarity.

Given the potential experimental artifacts with STEM-EELS, the quantification of the O concentration from XPS can be cited with more confidence. The maximum O concentrations measured at the interface from XPS depth profiling were 17 at % for the in situ pretreatment and 11 at % for the ex situ pretreatment, as shown in Figure 7b,d. In contrast, the PEALD-only AlF_3_ sample (no e-beam pretreatment) had a maximum of 35 at % O at the interface (Figure 6g). Thus, in agreement with the STEM-EELS results, XPS depth profiling also reveals that short e-beam plasma pretreatments overall reduce, but do not completely eliminate the interfacial O between our PEALD film and the Al mirror. In contrast, when longer e-beam plasma exposures are performed (no PEALD step), as shown in Section 3.2, the interfacial oxide is reduced to a much greater extent, i.e., only 1–2 at. % of O from XPS, which is close to the detection limit. The reason for this interfacial oxidation when combining the e-beam process with PEALD is still under investigation. However, it is possible that during the 10–30 min between the e-beam pretreatment and PEALD processes, oxygen from the surroundings (ALD reactor for in situ, atmosphere for ex situ) is getting to the surface and attacking the AlF_3_ formed by the e-beam plasma. In addition, the shorter e-beam plasma pretreatment resulting in thinner films (≈7 nm) may not be enough to fully convert the native oxide layer into AlF_3_ such that some of the native oxide is still present and can contribute to the interfacial oxygen seen after e-beam + PEALD processing. It should be noted that in the conversion/fluorination during the early stages of the e-beam plasma process, mass balance dictates that each formula unit of Al_2_O_3_ is converted into two formula units of AlF_3_, which translates to a volume expansion of approximately 2×.^52^ Thus, it is reasonable to assume that an AlF_3_ thickness of at least 2× the starting native oxide thickness is required before all the oxygen from Al_2_O_3_ is fully removed. Given native oxide thicknesses ranging from 4 to 7 nm for the Al mirrors used in this work, a considerable amount of oxygen could likely be expected from AlF_3_ films grown by e-beam plasma if their thickness is below 8–14 nm.

In view of these results, a second approach was pursued to prevent any native oxide formation when the Al mirror is exposed to the atmosphere. This method uses XeF_2_ exposure to passivate the surface of the Al mirror immediately after Al deposition and combines it with the use of either in situ or ex situ e-beam plasma as a pretreatment before PEALD of AlF_3_ (Strategies 6 and 7 in Table 1). Within 1–2 s of completing the Al deposition, the evaporative PVD chamber was filled with XeF_2_ to a partial pressure of 2–5 × 10^–4^ Torr for 3 min. This XeF_2_ exposure provides a very thin fluoride layer that should protect the Al surface from oxidation during atmospheric exposure once the mirrors are unloaded from the deposition chamber. To verify that the postdeposition XeF_2_ exposure sufficiently protects the mirror from initial oxidation, XPS of XeF_2_-protected mirrors that were kept in an N_2_-purged environment for two months showed that the surface contained <10 at % O. A bare Al mirror (no XeF_2_ protection) kept in a similar N_2_-purged environment shows an average of 40–45 at. % of O on the surface. These XPS results are plotted and shown in Figure S8.

By limiting the effect of initial oxidation from exposure to the atmosphere, these XeF_2_-protected mirrors help to determine if the e-beam and PEALD processes themselves are a source of oxygen. The same in situ and ex situ e-beam pretreatment followed by PEALD (detailed above) was performed on XeF_2_-protected mirrors. It is evident from the XPS depth profiles (Figure 8a,b) that both in situ and ex situ e-beam pretreatments followed by PEALD do not introduce oxygen into the interface. Using the XeF_2_-protected mirrors, which do not have a native oxide layer to begin with, an interface with 1.5–2.0 at. % of O is obtained with the full pretreatment and PEALD procedure.

**Figure 8:**
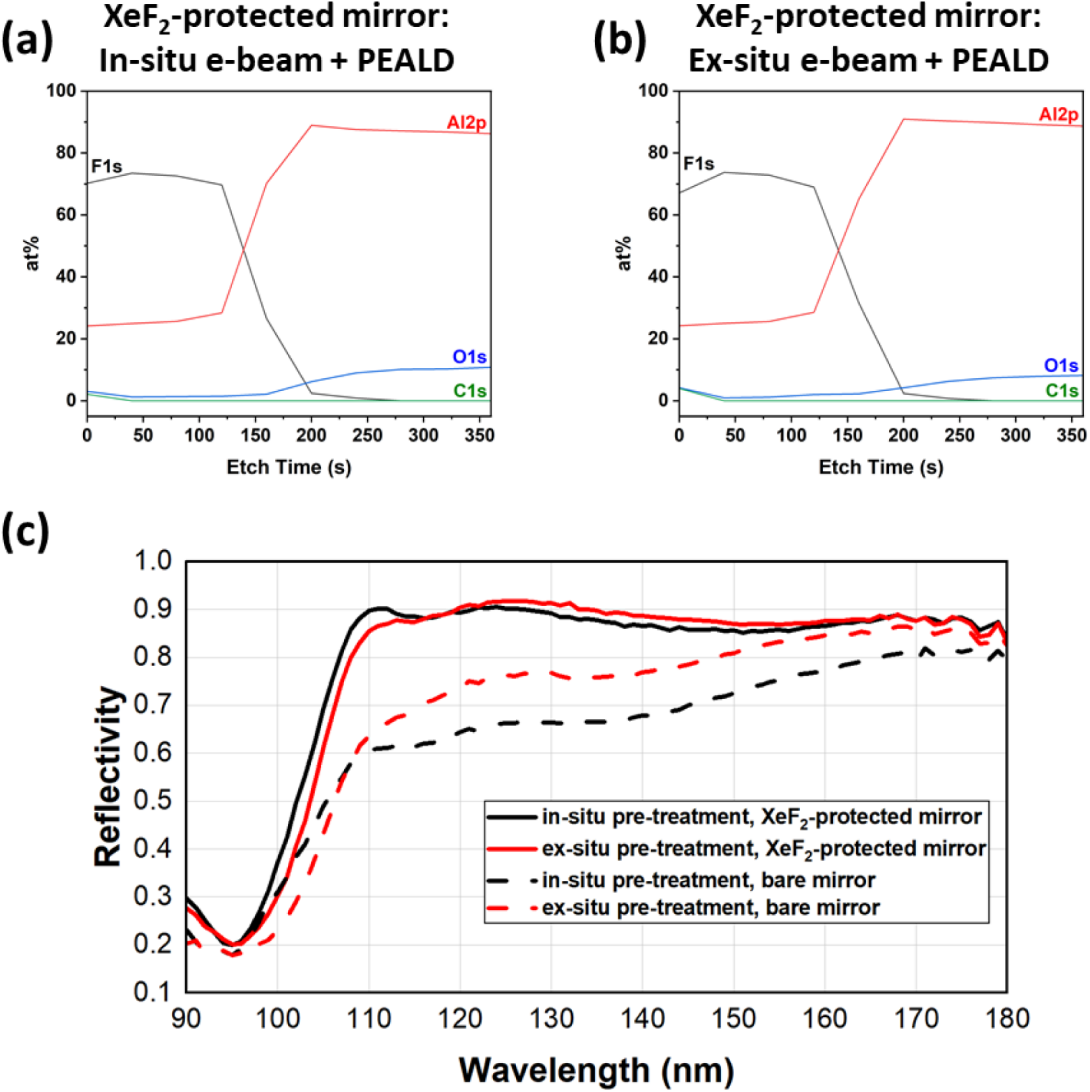
XPS depth profiles showing the relative concentrations of Al, F, O, and C species for processed XeF_2_-protected mirrors (Strategies 6 and 7 in Table 1): (a) in situ e-beam plasma pretreatment + PEALD AlF_3_ and (b) ex situ e-beam plasma pretreatment + PEALD AlF_3_. (c) Reflectivity as a function of the incident wavelength of the same samples. Dashed lines represent bare Al mirrors (no XeF_2_ exposure) that underwent the same in situ/ex situ pretreatment + PEALD.

As shown in Figure 8c (solid lines), the PEALD samples with combined XeF_2_ and e-beam pretreatments resulted in excellent reflectivity. These reflectivity curves are comparable to state-of-the-art e-beam plasma-processed mirrors, which have the best-reported reflectivity of AlF_3_-passivated Al to date.^36^ The reflectivity of e-beam pretreated, PEALD-coated mirrors that did not undergo prior XeF_2_ exposure (i.e., Strategies 4 and 5 in Table 1) is overlaid in Figure 8c (dashed lines) for comparison; these are equivalent to the samples whose XPS depth profiles are shown in Figure 7, which reveal some amount of interfacial O. As evident in Figure 8c, by limiting the starting native oxide on the mirror with the use of XeF_2_ exposure, reflectivity below 110 nm can be significantly improved for both the in situ and ex situ pretreatments. Moreover, for relevant spectral lines within the 100 to 160 nm range, the XeF_2_-protected mirrors show enhanced reflectivities of 43–52% at 102.6 nm (H Lyman β), 81–87% at 108.5 nm (He Lyman γ), 90–91% at 121.6 nm (H Lyman α), 89–91% at 130.4 nm (OI), and 86–87% at 155 nm (C IV). Overall, these results demonstrate that excellent reflective properties can be achieved using the passivation strategy of combining an e-beam plasma pretreatment with an ALD recipe for metal fluoride deposition.

Finally, all key results from the different passivation strategies, specifically the F/Al ratio of the film, interfacial O content, and reflectivity at 121.6 nm, are summarized in Table 3. It is worth noting that the F/Al ratios produced by all of the processes discussed in this work, ranging from 2.9 to 3.2, met the required stoichiometry of F/Al ≈ 3, and there is no correlation between the exact F/Al ratio in the film and the reflectivity of the mirror. Based on mirror performance at the 121.6 nm wavelength, strategies 1, 2, 6, and 7 provide the best reflectivity values. These four best-performing strategies were all processes that successfully limited the amount of oxygen at the AlF_3_/Al interface. Similar previous studies have shown that reducing the oxygen content within the AlF_3_ film consequently enhances FUV reflectivity,^37,53^ and this present study highlights the importance of reducing oxygen specifically at the interface region between the film and mirror. However, strategies 1 and 2, which employ e-beam plasma alone, can only yield AlF_3_ films on Al, whereas strategies 6 and 7 allow for more versatility in coating chemistry since the PEALD step can be tailored toward other fluoride materials as well.

**Table 3:** Summary of Results for All Passivation Strategies

	Processes included						
Strategy number	In-vacuo XeF_2_ passivation	In situ e-beam treatment	Ex situ e-beam treatment	PEALD	F/Al ratio in film	Interfacial O at % from XPS	Reflectivity at 121.6 nm
1		x			3.16	1 at %	0.92
2			x		3.17	2 at %	0.89
3				x	2.94	35 at %	0.67
4		x		x	2.94	17 at %	0.65
5			x	x	2.97	11 at %	0.75
6	x	x		x	2.93	2 at %	0.90
7	x		x	x	2.97	2 at %	0.91

## 4 Conclusions

In this work, Al mirrors for FUV applications are passivated with an AlF_3_ coating using a uniquely modified ALD reactor with a hybrid plasma system composed of remote ICP and e-beam-generated plasma sources. Different operating parameters for the e-beam plasma were explored, and optimal AlF_3_ properties were produced using a grounded sample surface, gas flows of 25/80 sccm SF_6_/Ar, and a substrate temperature of 150 °C. These optimal e-beam plasma operating conditions resulted in an abrupt AlF_3_/Al interface without an interfacial oxide layer, which was critical in achieving excellent reflectivity in the FUV range (i.e., 92% at 121 nm). This proved that the state-of-the-art e-beam plasma passivation technique, demonstrated in prior work in a dedicated e-beam plasma processing chamber (LAPPS), can be transferred to a growth-dedicated chamber such as an ALD reactor. This illustrates the potential of combining the benefits of e-beam plasma passivation and ALD for metal fluoride film growth.

This work then investigated different strategies to produce unoxidized Al surfaces for subsequent PEALD of metal fluoride thin films, such as AlF_3_. The strategies included the use of e-beam plasma as a pretreatment or prior in-vacuo XeF_2_ passivation combined with e-beam plasma. It was found that while the e-beam plasma and PEALD processes themselves do not introduce oxygen to the surface, a short e-beam plasma pretreatment was unable to fully remove the native oxide layer from the bare Al samples (Strategies 4 and 5). Thus, preventing the initial native oxide formation proved to be a crucial step in the overall passivation process. Initial XeF_2_ passivation was performed immediately after the fabrication of the Al mirrors, effectively inhibiting native oxide formation in the atmosphere between the different processing steps. Excellent reflectivity (90–91% at 121 nm and 43–52% at 103 nm wavelengths) was achieved by samples fabricated using the XeF_2_ + e-beam + PEALD technique reported here (Strategies 6 and 7).

## Supplementary Material



## Notes

Additional experimental details and supplementary AFM, STEM-EELS, and XPS results and analysis (PDF)
