# Supplementary material for: Passivation Strategies for Far-Ultraviolet Al Mirrors Using Plasma-Based AlF3 Processing
Source: Chem Mater. 2025 Sep 3;37(18):7450–61. doi: 10.1021/acs.chemmater.5c01881 (PMC12461835; doi:10.1021/acs.chemmater.5c01881)
Supplement: Supplementary file 1 [file cm5c01881_si_001.pdf]

Supporting Information

Passivation strategies for far-ultraviolet Al mirrors using plasma-based AlF_3 processing

*Maria Gabriela Sales [a], David R. Boris [b], Luis V. Rodriguez de Marcos [c], James L. Hart [d], Andrew C. Lang [b], Benjamin S. Albright [b], T. Jude Kessler [b], Edward J. Wollack [e], Manuel A. Quijada [e], Scott G. Walton [b], and Virginia D. Wheeler [b]**

[a] NRC Research Associateship Program, Washington, DC 20001, United States

[b] U.S. Naval Research Laboratory, Washington, DC 20375, United States

[c] Catholic University of America and NASA Goddard Space Flight Center (CRESST II agreement), Greenbelt, MD 20771, United States

[d] Nova Research Inc, Alexandria, VA 22308, United States

[e] NASA Goddard Space Flight Center, Greenbelt, MD 20771, United States

*Corresponding author: virginia.d.wheeler.civ@us.navy.mil

Effect of Negative Substrate Bias on the In-Situ E-Beam Plasma Process

To further understand the effect of ions arriving at the sample surface, e-beam plasma processing was performed while applying a negative substrate bias, which enhances the delivery of positive ions to the substrate. The bias voltage was varied from -10 V to -100 V at a constant gas flow of 25/80 sccm SF_6/Ar. Based on analysis of the resulting surface chemistry, as the magnitude of the bias voltage was increased, the F/Al stoichiometry increased, and the O content decreased (Fig. S1a). It is seen that -10 V and -30 V produced samples that were very F-deficient (F/Al = 0.99 to 1.75) with a high O concentration (15-25 at%), and a bias of -100 V was needed to achieve AlF_3 and suppress O contamination. While the oxide layer on the Al surface is still treated by the e-beam plasma, particularly at higher voltage, AlF_3 formation is not promoted or enhanced with a negative applied bias. This is evident in the X-ray photoelectron spectroscopy (XPS) depth profiles in Fig. S1b-d, where despite the reduction of the native oxide layer and incorporation of some amount of fluorine, all to varying degrees, there is no region demonstrating a fully formed AlF_3 film. While stoichiometry was good at -100 V, this film is comparatively thin compared to standard e-beam plasma AlF_3 samples shown in the main paper, which were processed with the sample surface electrically grounded.

Additionally, etching of the sample holder was observed with the applied substrate bias (Fig. S1e), which led to the presence of Pt contamination in our samples. Pt is used to coat our chamber walls and sample holder because it was found to be otherwise benign in SF_6/Ar plasma; however, the Pt coating on the sample holder is etched when a negative substrate bias voltage is applied. Therefore, in the main paper, samples were kept electrically grounded without a negative applied bias in order for the e-beam plasma treatment to take place effectively.

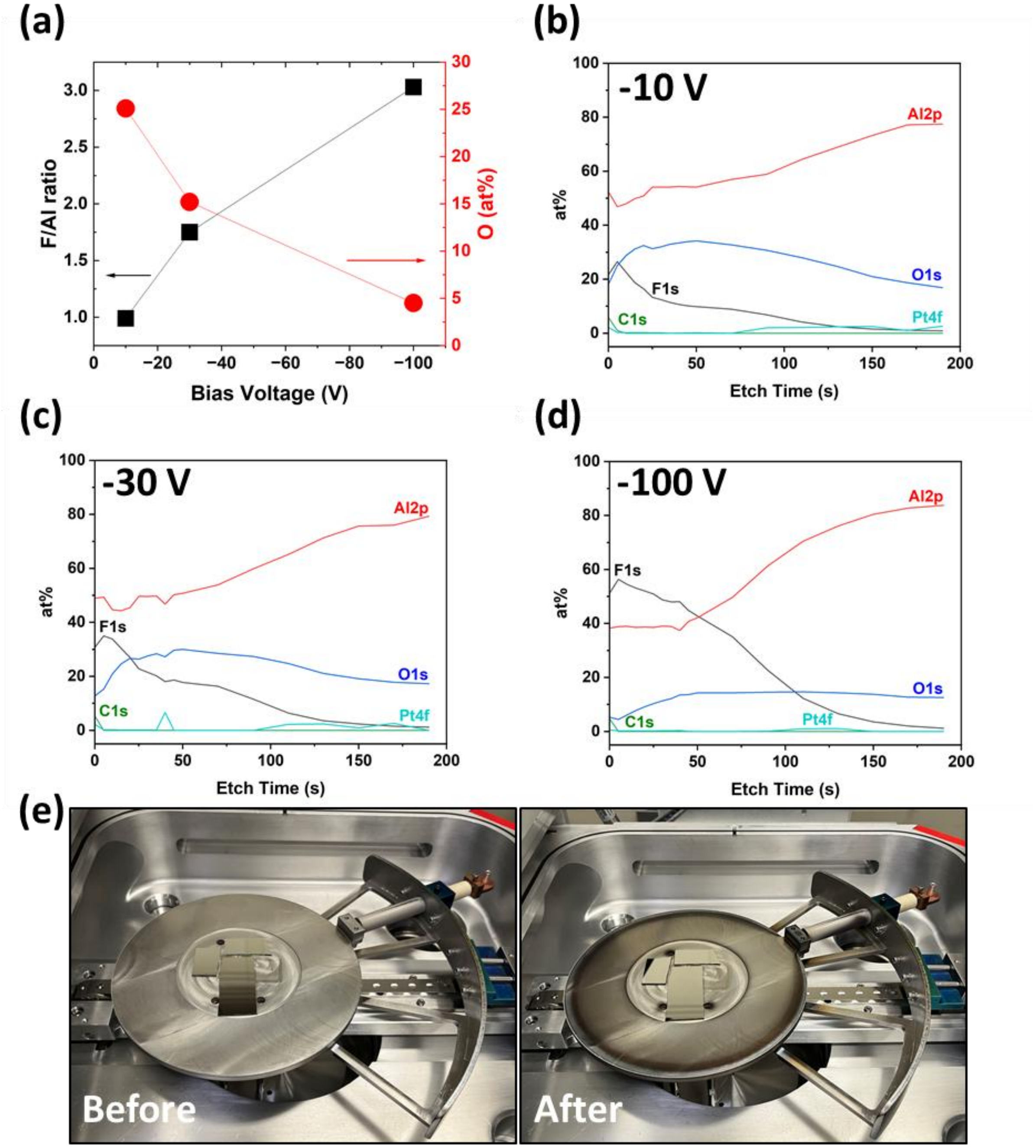

Figure S1. *(a) Quantified F/Al ratios and at% O as a function of negative applied bias voltage. XPS depth profiles showing the relative concentrations of Al, F, O, C, and Pt species in e-beam plasma-processed samples with a bias voltage of (b) -10 V, (c) -30 V, and (d) -100 V. The detected C is from adventitious surface contamination, and Pt is contamination from the ALD sample holder. (e) ALD sample holder before (left) and after (right) exposure to SF_6/Ar e-beam plasma with a negative applied substrate bias, wherein etching of the holder, especially at the edges, is evident.*

Atomic Force Microscopy (AFM) Results

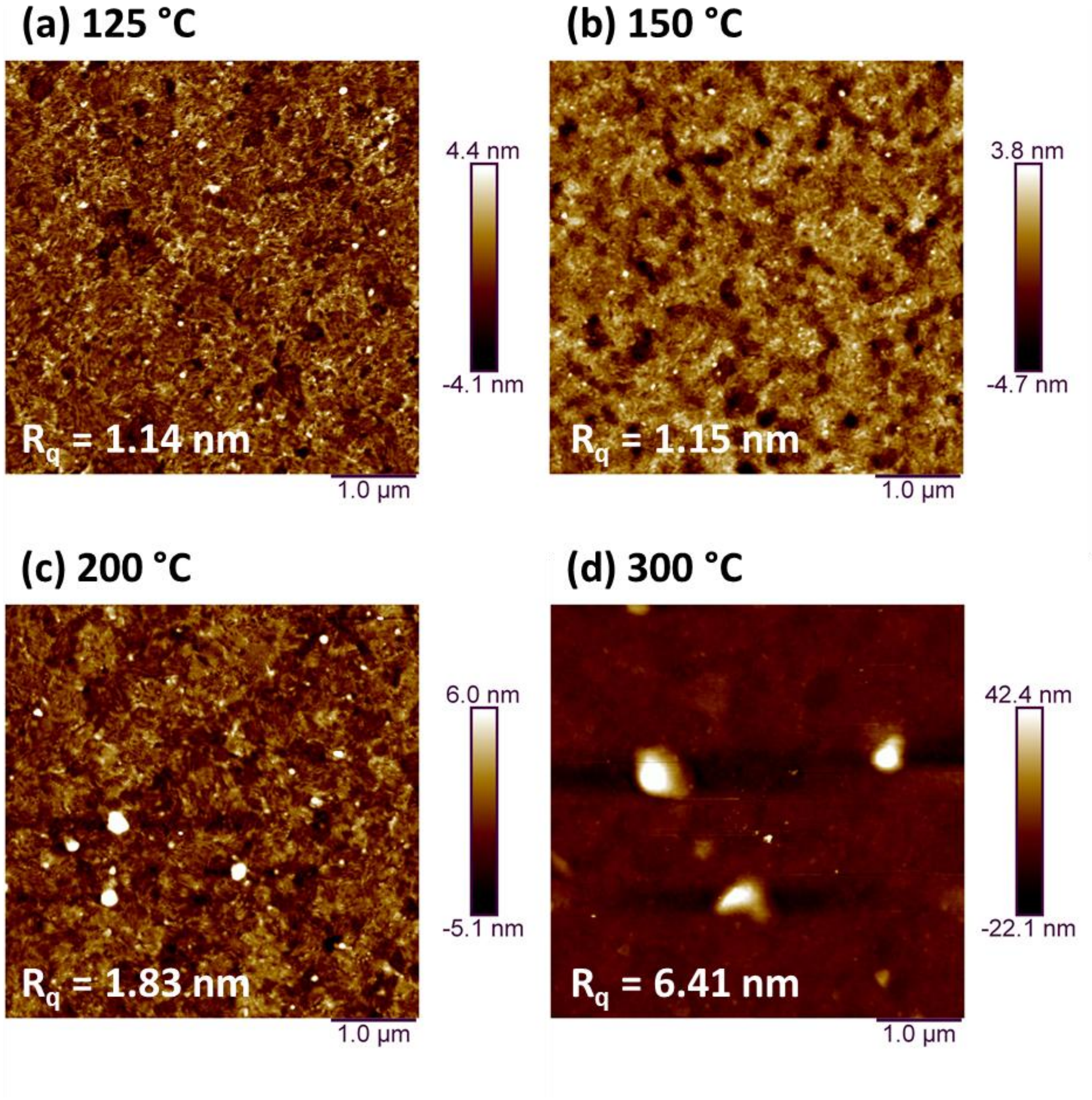

Figure S2. *AFM images of Al mirrors processed with* SF_6*/Ar e-beam plasma in the modified ALD reactor at (a) 125 °C, (b) 150 °C, (c) 200 °C, and (d) 300 °C.*

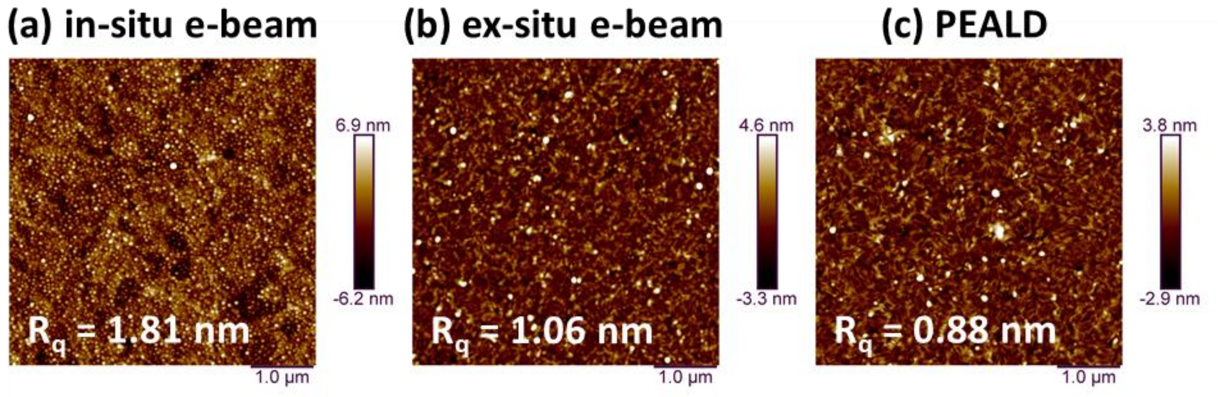

Figure S3. *AFM images of AlF_3-passivated Al mirrors processed using (a) in-situ e-beam plasma, (b) ex-situ e-beam plasma, and (c) PEALD. Strategies 1, 2, and 3 in main text; targeted AlF_3 thickness was 24-28 nm.*

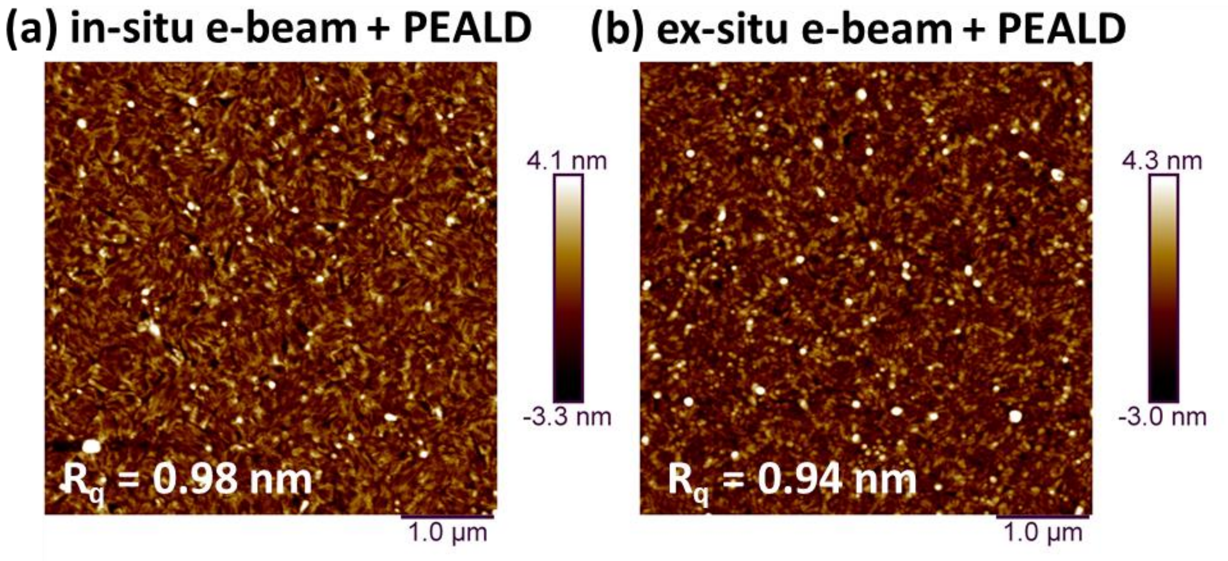

Figure S4. *AFM images of PEALD AlF_3 films on Al mirrors that underwent (a) in-situ e-beam pre-treatment and (b) ex-situ e-beam pre-treatment. Strategies 4 and 5 in main text; targeted AlF_3 thickness was 24 nm.*

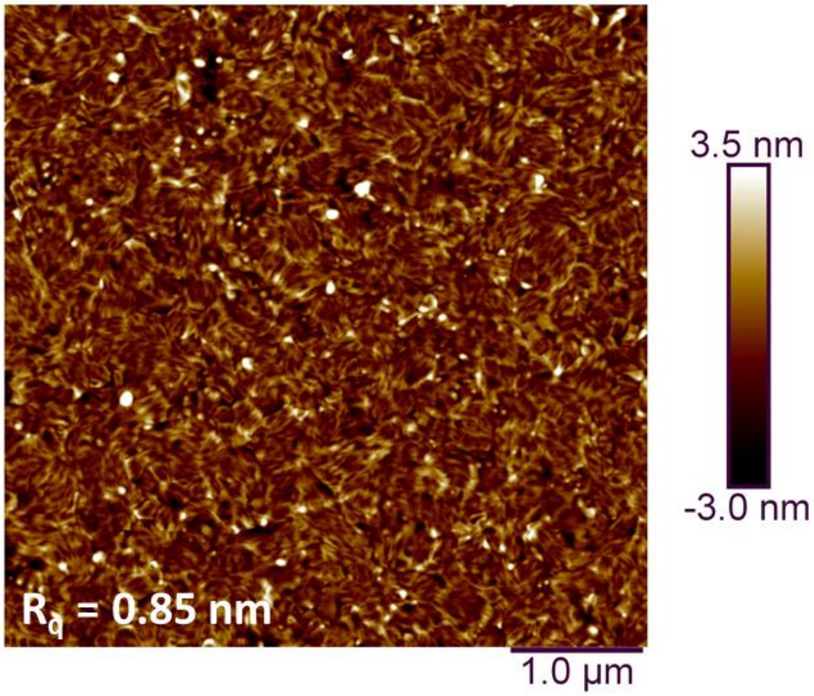

Figure S5. *AFM image of a bare as-deposited Al mirror for reference.*

Thickness and Oxidation Effects with Scanning Transmission Electron Microscopy Electron Energy Loss Spectroscopy (STEM-EELS)

Focused ion beam (FIB) sample preparation creates new surfaces. If these newly created surfaces oxidize before the sample is placed in the STEM, then the surface oxide will influence composition determination. The relative influence of the surface oxide will depend on the thickness of the STEM lamella (which determines the surface-to-volume ratio). Note that the relevant thickness is not the thickness of the AlF_3 film; rather, it is the thickness of the FIB lamella, which depends on the FIB preparation process and is typically 10-100 nm. The FIB lamella thickness can also vary across a given sample. Thus, by measuring the same sample in different locations with different thicknesses, it is possible to plot the oxygen content as a function of sample thickness. This data allows the determination of surface oxidation effects.

Figure S6 shows the oxygen composition in atomic % measured for five different samples. For each sample, measurements were collected at various regions of the sample with different local thicknesses. The sample thickness is extracted from the EELS measurements, which gives the thickness in units of inelastic mean free paths (MFPs). Figure S6a shows the interfacial oxygen content versus the local sample thickness. Figure S6b shows the oxygen content within the interior of the AlF_3 film (excluding the Al interface and the protective carbon interface) versus the local sample thickness. For both plots, the measured amount of oxygen decreases for thicker samples. This effect is due to surface oxidation, as discussed above. The most reliable oxygen values come from the thicker regions of the specimen. When comparing the oxygen content from STEM-EELS between different samples (see Fig. 6 and 7 of the main text), it is important to make the comparison at a similar thickness. As shown in Figure S6a, for any given sample thickness, PEALD only has the highest interfacial oxygen contamination.

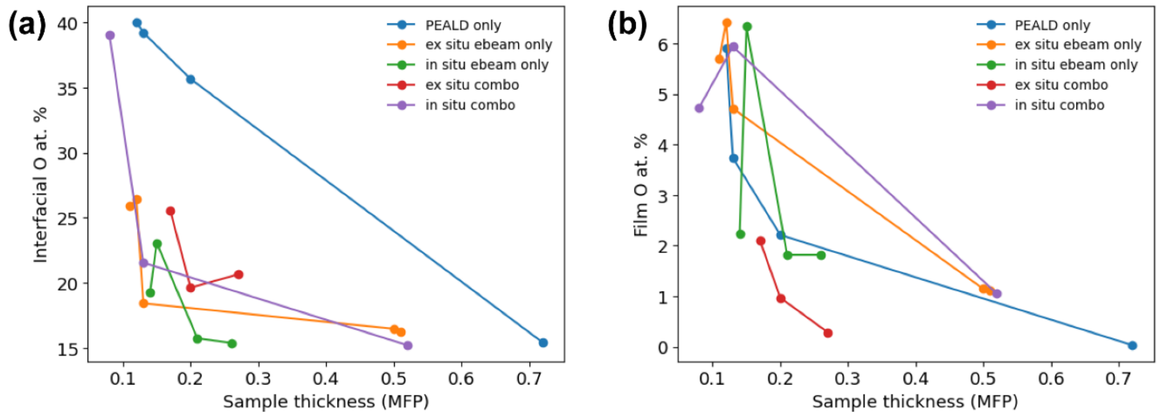

***Figure S6**. STEM-EELS oxygen composition determination as a function of local sample thickness for five different AlF_3-passivated Al mirrors. The oxygen at% at the AlF_3/Al interface is given in (a), and the oxygen at% within the AlF_3 film interior is given in (b).*

AlF_3 Sample Damage During STEM Measurements

The sample damage observed during STEM imaging is demonstrated in Fig. S7. At low dose imaging conditions (200 kV, < 10 pA, 2 ms dwell time, 1.5 nm pixel size, ~500 e/Å^2 per pass), there is no visible damage to the AlF_3 film after a single pass via STEM annular dark-field (ADF) imaging. After a second pass, damage is observed, primarily at the top and bottom of the film, with further damage being visible after a third pass. The damage is also observed via STEM-EELS mapping of the Al plasmon at ~15 eV. The plasmon maps show the presence of a few small metallic Al islands after the first pass, and an increased number of islands generated after the second pass.

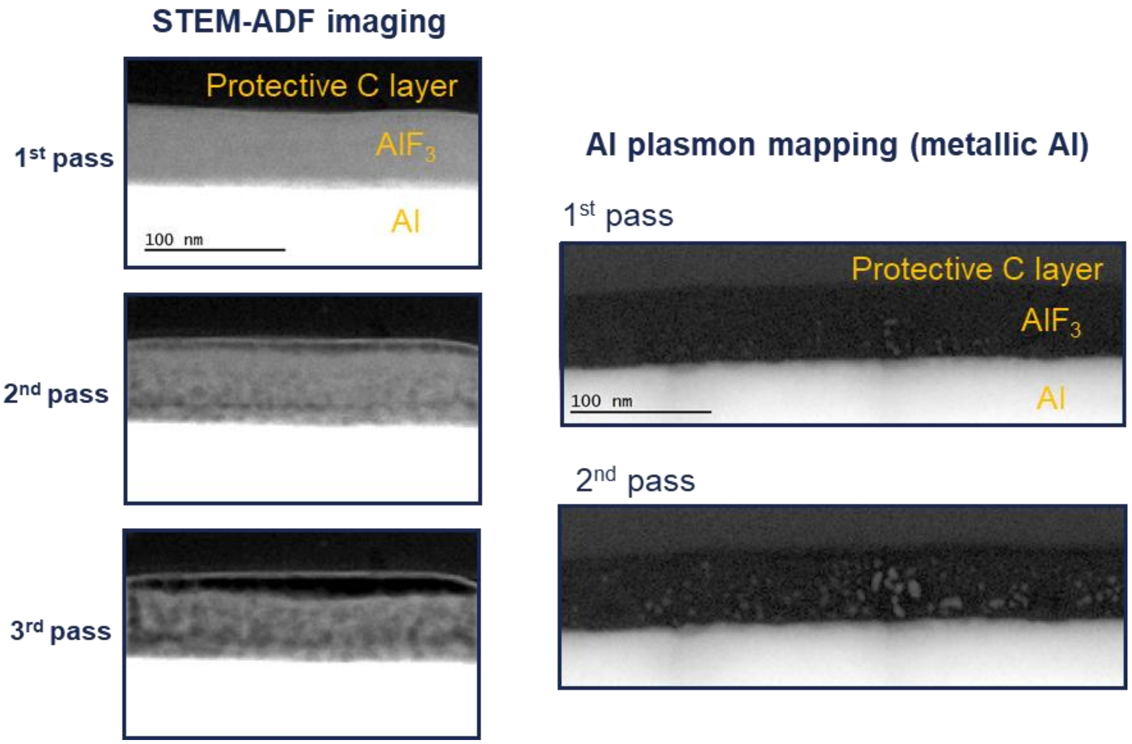

Figure S7. *STEM-ADF cross-sectional imaging of an* AlF_3 *film on an Al mirror substrate after sequential passes (left). Mapping of Al plasmon features showing the presence of metallic Al (bright regions) within the* AlF_3 *layer after two passes (right).*

XPS Depth Profiles of Al Mirror Substrates

Approximately two months after Al mirror deposition, during which these samples were stored in N_2-purged containers, XPS depth profiling was performed to investigate the chemical composition at the surface and in the bulk of the Al films (Fig. S8). A bare Al mirror had 40-45 at% O on the surface, while a mirror that underwent in-vacuo XeF_2 passivation immediately after Al mirror deposition only had < 10 at% O on the surface after two months. The XeF_2 passivation fluorinates the top surface of the Al, forming a very thin surface AlF_3 layer that then protects the Al mirror from native oxide formation.

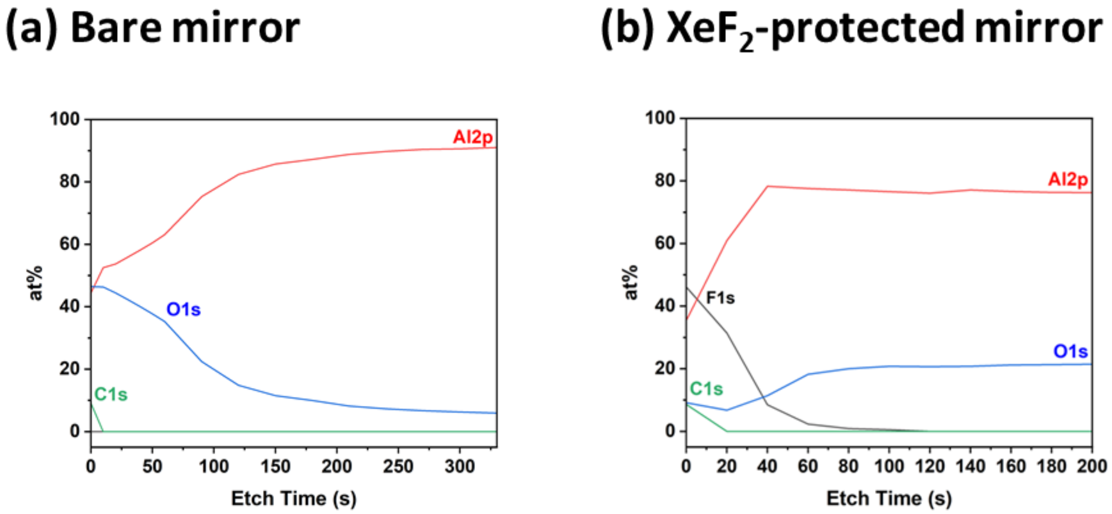

Figure S8. *XPS depth profiles showing the relative concentrations of Al, O, C, and F species in (a) a bare Al mirror substrate, and (b) an XeF_2-protected Al mirror substrate. XPS measurements were performed after approximately two months of storage in N_2-purged environments.*
